# Does Gut-Microbiome Interaction Protect against Obesity and Obesity-Associated Metabolic Disorders?

**DOI:** 10.3390/microorganisms9010018

**Published:** 2020-12-23

**Authors:** Agnieszka Zawada, Anna Maria Rychter, Alicja Ewa Ratajczak, Agata Lisiecka-Masian, Agnieszka Dobrowolska, Iwona Krela-Kaźmierczak

**Affiliations:** Department of Gastroenterology, Dietetics and Internal Diseases, Poznan University of Medical Sciences, 49 Przybyszewskiego Street, 60-355 Poznan, Poland; aga.zawada@gmail.com (A.Z.); alicjaewaratajczak@gmail.com (A.E.R.); agata.lisieckamasian@gmail.com (A.L.-M.); agdob@ump.edu.pl (A.D.); krela@op.pl (I.K.-K.)

**Keywords:** gut microbiota, chronic diseases, obesity, diabetes, diet

## Abstract

More research has recently focused on the role of the gut microbiota in the development or course of numerous diseases, including non-communicable diseases. As obesity remains prevalent, the question arises as to what microbial changes are associated with increased obesity prevalence and what kind of prevention and treatment approaches it could provide. Moreover, the influence of the gut-brain axis on obesity is also crucial, since it can affect metabolism and food intake. The quantitative and qualitative changes in the microbiota composition are called dysbiosis; however, in view of the current knowledge, it is difficult to conclude which microbial imbalances are adverse or beneficial. Increased numbers of pathological microorganisms were observed among patients with obesity and comorbidities associated with it, such as diabetes, cardiovascular disease, and insulin resistance. Our review provides current knowledge regarding changes in the intestinal microbiota associated with obesity and obesity-associated comorbidities. Nevertheless, given that dietary patterns and nutrients are two of the factors affecting the intestinal microbiota, we also discuss the role of different dietary approaches, vitamins, and minerals in the shaping of the intestinal microbiota.

## 1. Introduction

A microbiota is a complex and dynamically developing ecosystem, which is continuously changing during a lifetime. It consists of commensal, symbiotic, and pathogenic microorganisms. The microbiota has the attributes of climax moves toward homeostasis and, as much as it has been discovered, is unique for every individual. Around 30% remains a sort of “species-core”; however, the rest of the species could be subjected to both qualitative and quantitative changes influenced by various factors, either intentional or not [1,2]. Moreover, these factors could be individual-independent and associated with genetic, or environmental factors as well as with the type of birth [3,4]. On the other hand, microbiota changes could be connected with modifiable aspects, such as diet (including prebiotics and xenobiotics), or the application of probiotics. According to the research, different and the specific microbiota compositions have been found in metabolic, neurologic, and functional disorders, with more research being conducted to investigate the role of the intestine microbiome in the development of type 2 diabetes (T2DM), obesity, and metabolic disorders. Not only are these research studies focused on the quality and quantity of the intestinal microbiota and its influence on the body weight, but they also investigate the association between the diet and its influence on the microbiota composition. Additionally, the microbiota–gut–brain axis could be another possible pathway linking the intestinal microbiota with metabolism, and this bidirectional system can influence both host metabolite and appetite.

As the number of people suffering from excessive body weight increases, obesity has become one of the most prevalent diseases worldwide, regardless of sex or age. Additionally, according to the World Health Organization (WHO), the prevalence of obesity has tripled since 1975. As the WHO’s report highlighted, even though obesity is preventable and different approaches are proposed by various specialists, its prevalence is continuously increasing. The comorbidities associated with it (e.g., T2DM and cardiovascular diseases (CVD)) are the cause of millions of deaths each year. In fact, almost 40% and 13% of people above 18 years of age are overweight and obese, respectively [5], with most of the world’s population living in countries where overweight and obesity kill more people than underweight.

It remains to be found whether and to what extent the intestinal microbiota could be associated with increased obesity prevalence. It has been suggested that individuals with lower amount of gut microbial genes and lower bacterial richness have increased risk of type 2 diabetes and obesity [6,7]. Dysbiosis among patients with T2DM promotes pro-inflammatory signalling, which induces metabolic syndrome [8]. In recent years, many studies have shown that diet, particularly a high-fat diet (HFD), significantly changes the number of *Bacteroidetes* species and increases the number of *Firmicutes* and *Proteobacteria* species. Turnbaugh et al. were the first to discover that the contribution of *Bacteroidetes* is substantially smaller (around 20%) in obese mice than in lean mice (around 40%) [9]. In addition, Flessner et al. showed that implementing a high-fat and low-fibre diet is associated with decreased Bacteroidetes and increased *Firmicutes*. Moreover, transplantation of the microbiota from obese to germ-free mice is associated with significant adipose tissue deposition, compared with transplantation from leaner mice but with the same diet [10]. On the other hand, changing the diet from low- to high-fat is associated with a decrease of *Bacteroidetes* in the intestines and an increase in *Firmicutes* and *Proteobacteria*, regardless of the body weight [11]. Additionally, it has been proven that a high-protein and low-carbohydrate diet often leads to the quantitative deficiency of *Bifidobacterium* in the intestinal tract. It is generally known that the intestinal microbiota plays a significant role in the digestive process; however, it is unknown what composition of the intestinal microbiota protects and what type promotes the development of metabolic disorders [12]. Thus, it could be suggested that dysbiosis could be regarded as another risk factor for various diseases, including metabolic disturbances. Therefore, restoring gut composition could provide an essential value for their further treatment [13].

## 2. The Microbiota–Gut–Brain Axis

**Figure 1:**
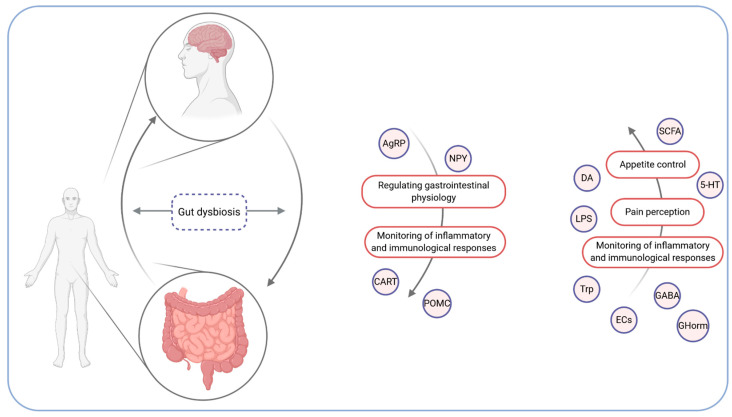
The brain–gut axis. AgRP—agouti-related protein; CART—cocaine- and amphetamine-regulated transcript; POMC—pro-opiomelanocortin; NPY—neuropeptide Y; GHorms—gastrointestinal hormones (e.g., cholecystokinin, leptin, glucagon-like peptide 1); DA—dopamine; SCFAs—short-chain fatty acids; 5-HT—5-hydroxytryptamine; LPS—lipopolysaccharide; Trp—tryptophan; ECs—endocannabinoid ligands; GABA—gamma-aminobutyric acid.

The gut–brain axis is a bidirectional communication system involving neural, endocrine, metabolic, and immunological signalling. The afferent path of the gut to the brain is essential for appetite control (food intake and satiety), the perception of pain, and the brain’s monitoring of the gut’s inflammatory or immunological status [14]. In return, the brain regulates gastrointestinal physiology and, by means of such elements as vagal afferents, modulates inflammatory and immunological responses [15]. It includes the central nervous system (CNS), the enteric innervation, including extrinsic fibres of the autonomous nervous system (ANS), HPA axis (hypothalamic–pituitary–adrenal axis), intrinsic neurons of the enteric nervous system (ENS), and the intestinal microbiota [16]. HPA axis regulates body processes, including digestion, and releases the corticotrophin-releasing factor, which affects intestinal inflammation, permeability, and motility [12]. The CNS and the ENS are associated with gastrointestinal tract functioning. Changes in eating behaviour in response to the CNS’s appetite control can affect the availability of nutrients for intestinal microbiota and further affect its composition. On the other hand, the intestinal microbiota can modify satiety signals by producing proteins, such as the α-melanocyte-stimulating hormone [17]. The mechanism of the brain-gut axis is depicted in Figure 1.

The integration of the microbiota into this axis (microbiota–gut–brain axis) is based on the afferent component where the microbiota can affect the brain and behaviour, and descending pathways where alterations in brain function can affect changes in the microbiota composition [18]. In fact, the microbiota–gut–brain axis could be another possible pathway linking the intestinal microbiota and metabolism. However, knowledge about the influence of the intestinal microbiota on the gut–brain axis is limited—it can affect the brain indirectly by gut-derived neuronal, immune, and neuroendocrine molecules affecting spinal and vagal afferents. On the other hand, the gut microbiota can additionally communicate with the brain directly by microbe-generated signals [13]. It is essential to notice that any perturbations, including intestinal dysbiosis, can affect the gut–brain axis manifesting in brain–gut disorders [19]. Gut microbiota-derived short-chain fatty acids (SCFAs)—indispensable for the synthesis of lipids and glucose—can influence both host metabolite and appetite; nevertheless, the likely underlying mechanisms have not been fully understood [18]. Moreover, the intestinal microbiota can induce inflammation by releasing lipopolysaccharide (LPS), namely an enterotoxin produced by an external cell membrane of cyanobacteria and Gram-negative bacteria, which induces immune cell activation and cytokine production [20,21]. Neuroendocrine signalling pathways between the intestinal microbiota and the brain are associated with microbiota-derived neuroactive metabolites, such as dopamine, gamma-aminobutyric acid, tryptophan, endocannabinoid ligands, and 5-hydroxytryptamine (serotonin precursor), which centrally and peripherally affect the host’s metabolism by the vagal stimulation, or immune neuroendocrine mechanisms [22]. Moreover, the intestinal microbiota can communicate with the enteric innervations by such means as an interaction with immune cells and enteroendocrine cells (EECs), which, through the activation of different receptors, produce hormones secreted by gastrointestinal tract hormones in response to a bacterial inducement [23]. Hormones like cholecystokinin (CCK), leptin, glucagon-like peptide 1 (GLP-1), glucagon-like peptide 2 (GLP-2), peptide YY (PYY), and ghrelin trigger the vagal and spinal nerves [24]. Furthermore, signals are integrated in the hypothalamus responsible for the regulation of energy metabolism. In this area, neurons, such as agouti-related protein (AgRP) and NPY (neuropeptide Y), increase appetite and lower energy expenditure (through melanocortin and orexin). However, neurons, such as CART (cocaine- and amphetamine-regulated transcript) and POMC (pro-opiomelanocortin), work in the opposite direction and are associated with increased energy expenditure and inhibited food intake (through the α-melanocyte-stimulating hormone) [25].

## 3. The Mechanism of the Microbiota Influence on the Development of Obesity and Its Comorbidities

It is not necessarily clear whether the intake of specific nutrients supports or inhibits the growth of certain types of bacteria, as well as whether it is a direct cause of weight gain. It seems that in the process of digestion and energy absorption, diversity and the ability to inhibit pathogens’ growth are essential in the complex synergy of the microbiota and human organisms. Several mechanisms could explain the association between the microbiota and human metabolism as well as its influence on the development of obesity. The intestinal microbiota, via the secretion of numerous chemical compounds, can increase the capillary refill density in the epithelium and lead to increased absorption of monosaccharides [26]. On the other hand, the symbiosis between the human organism and the microbiota allows for the fermentation of generally non-fermentable—by human enzymes—compounds, which can further provide an additional energy source; for instance, these compounds include SCFAs, such as acetate, butyrate, and propionate [27]. Another concept is that intestinal dysbiosis affects the proper synthesis of zonulin and occludin, which constitute the crucial aspects of the structural junction providing the integrity of enterocytes. Furthermore, dysbiosis leads to deterioration in the intestinal mucosa, which causes the “leaking” of various antigens and other harmful substances, mainly lipopolysaccharide. Since the substances “leak” through the intestinal barrier, they cause chronic inflammation, hence negatively affecting the entire metabolism. Additionally, LPS is associated with the muscle’s inability to dispose of glucose, which leads to the development of non-alcoholic fatty liver disease and insulin resistance and increases macrophages infiltration in the adipose tissue. Additionally, both SCFAs and LPS stimulate the excretion of PYY, slowing down the digestive tract motility and, consequently, affecting the absorption of essential nutrients. The excessive excretion of the hormone-like peptide may be associated with the development of obesity, mainly since it has been observed that people suffering from obesity present higher concentrations of faecal SCFAs [28]. However, butyrate’s energy homeostasis activity is different, since it stimulates the secretion of leptin in the adipocytes and induces the secretion of GLP-1 in the epithelium. It also affects both the oxidation of fatty acids and the activity of the mitochondria in the muscle and the brown adipose tissue, which results in increased thermogenesis. Studies of a model of insulin-resistant obese mice fed with a high-fat diet enriched in butyrate showed an increase in insulin sensitivity. Furthermore, it was observed that mice fed with a low-carbohydrate diet presented lower butyrate concentration and butyrate-producing bacteria, leading to the conclusion that butyrate alone is beneficial to metabolism in pathological conditions, although it does not play a major role in normal conditions [20]. Both LPS and SCFAs can induce the accumulation of the adipose tissue due to the inhibition of the fasting-induced adipose factor (FIAF). FIAF, in turn, inhibits the action of lipopolysaccharide lipase responsible for the energy storage in the adipose tissue. On the other hand, the intestinal microbiota can affect lipid metabolism due to the inhibition of protein kinase activity activated by AMP, which controls the energy status at the cellular level. Research on germ-free mice demonstrated that despite administering a high-fat and high-carbohydrate diet, individuals with a high activity of phosphorylated AMP in the muscles and liver (high efficiency of the oxidation of fatty acids in those organs) did not develop obesity [29].

Another hypothesis assumes that the microbiota influences the pathomechanism of obesity by means of storing bile acids, as well as by their composition. One of the roles of bile acids is to activate the receptors affecting glucose and lipids’ metabolism. The FXR (farnesoid X receptor), a nuclear receptor, and the TGR5 (G-protein-coupled bile acid receptor), a membrane receptor, are particularly relevant in this process. The FXR was the first identified bile-stimulated receptor. A study on FXR-free mice revealed higher serum concentrations of triglycerides and glucose than in mice with a proper activation of the FXR. Hence, since the activation of the TGR, which is located in the brown adipose tissue and small intestine, increases the concentration of cAMP, it also leads to higher energy use in the brown adipose tissue. Therefore, it could be responsible for the lower incidence of obesity and insulin resistance [30,31].

## 4. The Microbiota and Body Weight Reduction

### 4.1. Probiotics

The effectiveness of probiotic use among patients with obesity remains controversial. On the one hand, probiotics have been used for years both in animal husbandry to increase animals’ body weight and among infants to support their growth and the development of the organism, since probiotic administration can affect energy recovery from food and its storage [32]. In 2012, Million et al. conducted a meta-analysis on the influence of lactic acid bacteria on weight [33] leading to an observation that bacteria from one species affect body weight differently (Table 1) [33].

Additionally, the results showed that the probiotic effect is specific for each species and should be followed by clinical trials. Similar results were observed in a study by Crovesty et al. It has been highlighted that the use of *Lactiplantibacillus plantarum* and *Lacticaseibacillus rhamnosus* combined with a hypocaloric diet can result in body weight reduction [34]. However, other studies have shown that many probiotic species can be successfully used in the treatment of metabolic disorders (Table 2) [35].

The probiotic species can indirectly influence the improvement of intestinal tightness and limit the development of intrasystemic toxaemia, which is associated with the growth of pathogenic microorganisms. Consequently, the inflammation decreases, and insulin sensitivity, lipid profile, and carbohydrate metabolism undergo stabilisation. However, the data on probiotic use among patients with obesity are inconclusive. In a meta-analysis, probiotic use did not affect body weight reduction significantly [36]. Similar results were obtained in another meta-analysis where the probiotic use was associated with a decreased body weight and adipose tissue percentage; however, the results were not statistically significant when compared with the placebo group [37].

### 4.2. Prebiotics

The use of prebiotics also plays a role in the treatment of obesity. The presence of prebiotics in the intestine is associated with the production of both protective mucin and short-chain fatty acids as well as the production of anti-inflammatory cytokines in Peyer’s tufts. Additionally, prebiotics are related with the production of GLP-1 and GLP-2, which are essential in the regulation of lipid and carbohydrate metabolism. Interestingly, prebiotics are associated with the secretion of satiety hormones and, therefore, prevent excessive eating. A randomised, controlled trial on patients with obesity indicated that prebiotic-only supplementation resulted in a decrease in lipid profile and HbA1c and the improvement of anthropometric measures (body weight and trunk adipose tissue content). According to a study by Delzenee et al., prebiotics also affect carbohydrate metabolism. It has been shown that an oligofructose-enriched diet is associated with an increase in GLP-1 and its proglucagon precursor mRNA in the proximal part of the colon [38]. What is more, it has been observed that an increase in portal vein concentrations of GLP-1 and peptide YY (PYY) decreases ghrelin and appetite [38]. Additionally, prebiotics could be useful in the regulation of the intestinal endocannabinoid system [39] and increase the number of *Lactobacillus* and *Bifidobacterium.* Moreover, since they increase the synthesis of tight-junction proteins (zonula occludens 1 and 2), prebiotics are associated with improvement in the function of the intestinal barrier [40]. In fact, they decrease the inflammatory status by decreasing the concentration of pro-inflammatory cytokines and are associated with lower cardiovascular risk [41].

### 4.3. Synbiotics

According to Ferrareze et al., the supplementation of synbiotics (including *Lactobacillus gasseri*) is associated with a body weight reduction and has anti-inflammatory properties. Studies employing glucomannan and inulin fibre, favourable in the production of SCFAs, demonstrated a higher body weight reduction and gut microbiota reconfiguration. Novel synbiotics can reduce the amount of visceral fat area (VFA) and, therefore, can decrease insulin resistance and cardiovascular risk and the risk of developing T2DM [42]. Due to the fact that they influence changes in the microbiota composition, it seems that the optimal effects of the obesity treatment can be achieved by the supplementary use of synbiotics, as they can induce a more significant body weight reduction among patients suffering from obesity than among non-supplemented individuals [33].

## 5. The Microbiota and Fatty Liver Disease

In view of the frequent prevalence of obesity, non-alcoholic fatty liver disease (NAFLD) has become one of the most common chronic comorbidities associated with obesity. NAFLD’s pathological spectrum is extensive—from non-alcoholic steatohepatitis (NASH) through fibrosis and subsequently to cirrhosis and cancer. Besides the diet or genetic polymorphisms, the microbiota can also affect the course of liver disease with a similar mechanism as for diabetes or obesity [43]. Inappropriate dietary habits can lead to increased intestine permeability and small intestine bacterial overgrowth (SIBO). In a study by Miele et al., NAFLD was associated with increased permeability and SIBO, which simultaneously are markers for the severity of hepatic steatosis. Increased levels of LPS, NF-kB, and TNF-alpha have also been associated with liver malfunction [44]. Moreover, bacterial translocation increases the infiltration of endotoxins into the portal vein and decreases FIAF concentrations. Additionally, they increase the activity of the lipoprotein lipase, which supports the de novo synthesis of fatty acids and triglycerides and activates inflammatory toll-like receptors in the hepatocytes [29,45]. What is more, patients suffering from obesity with NASH present a decreased number of *Faecalibacterium* [46], which possesses anti-inflammatory properties due to the production of NF-kB and interleukin-8 (IL-8) inhibitors and acts locally in the colon by means of anti-inflammatory cytokine induction [47]. On the other hand, *Ruminococcus* is associated with increased production of SCFA, which promotes NAFLD development [48]. The data concerning *Lactobacillus* are inconclusive—it is a complex strain, and its metabolic function depends on the species [49]. In fact, some species can produce lactic acid from dietary carbohydrates, which further produces acetate and ethanol associated with liver dysfunction [50]. According to Durante et al., an increased level of *Lactobacillus* among patients with obesity, compared with their leaner count partners, is associated with a more severe liver cirrhosis course [51], and a similar result has been observed with *Bacteroides* and *Escherichia.^.^* In contrast, increased levels of *Bifidobacterium* spp. and *Akkermansia muciniphila* are negatively associated with adipose tissue inflammation and the levels of glucose, insulin, and triglycerides [52]. Prebiotic and symbiotic supplementations regulate the expression of genes associated with oxidisation (PPAR-alpha receptors) and lipogenesis (protein SREBP-1c), which further result in smaller cholesterol-dependent changes in the liver [53]. According to the study by Xu RY et al., probiotic supplementation (*Lactobacillus* and *Bifidobacterium*) lowered the liver’s fat accumulation among rats fed with a high-fat diet. However, no significant changes in intestinal permeability were observed when probiotic rats were compared with rats in the control group [54]. Furthermore, faecal microbiota transplantation (FMT) could improve NAFLD treatment, since FMT was associated with a lower fat accumulation in the liver and lower insulin resistance of the tissues in the animal models. A decrease of pro-inflammatory cytokines was also observed [55]. It is vital to bear in mind that insulin resistance constitutes one of the leading causes of not only T2DM but also obesity-associated NAFLD. The extensive accumulation of triglycerides in the liver can result in the peroxidation of lipids and the accumulation of reactive oxygen species, which delays the inflammatory response and leads to liver cirrhosis. Nevertheless, due to the reduction of insulin resistance, the administration of the novel antidiabetic drugs can improve the course of NAFLD [56].

## 6. Type 2 Diabetes and Changes in the Microbiota Composition

Type 2 diabetes mellitus (T2DM) is a metabolic disease associated with an impaired fasting and post-meal concentrations of glucose. Moreover, it is usually associated with the presence of obesity and dyslipidaemia. These disorders are mainly associated with a higher energy intake, including energy from xenobiotics which is essential for maintaining a proper health status. As it has already been mentioned, the aforementioned factors affect the composition of the microbiota. In fact, the correlation between T2DM and the microbiota is mainly associated with obesity-related mechanisms. As a result of T2DM, decreased levels of butyrate-producing *Firmicutes*, *Roseburia intestinalis*, and *Faecalibacterium prausnitzii* and increased levels of the pathogenic microorganisms were observed [57,58]. Additionally, the increased intestine permeability and the loosen up of the tight junctions could also result from both T2DM and the insulin resistance. Moreover, insulin-resistant individuals present an increased capacity to synthesize the branched-chain amino acids (BCAA) and a decreased capacity of the BCAA transport into the bacterial cells. The extensive BCAA production is associated with the presence of *Prevotella copri* and *Bacteroides vulgatus*. Other studies also confirmed that the intestinal microbiota has been associated with increased BCAA levels among insulin-resistant individuals [59]. Zhang et al. confirmed that decreased *A. muciniphila* have been associated with a higher risk of developing carbohydrate-metabolism disorders, as patients with pre- or newly diagnosed diabetes had smaller amounts of those bacteria. Interestingly, it has been suggested that the amount of *A. muciniphilia* can be a specific marker for the presence of impaired glucose tolerance [57]. Moreover, the presence of prediabetes and T2DM has been associated with a higher number of *Betaproteobacteria*. Additionally, T2DM had a lower number of *Faecalibacterium prausnitzii. F. prausntizii* are also administered in the FMT to reduce the inflammatory state and type 2 diabetes risk [60]. Another significant aspect of T2DM association with the microbiota composition is the type of ordered treatment. Metformin, i.e., the first-line treatment, has been considered a promising lead in the intestines’ pharmacological treatment, including changes in the microbiota [61]. In the study by Shin et al., a 6-week treatment with metformin resulted in changes in the microbiota among obese mice [62]. Furthermore, according to Lee et al., metformin improved both the metabolic disorders and the microbiota composition in the hypercholesterolemic mice [63]. As the observational studies have shown, metformin use promotes the growth of butyrate-producing bacteria and increases the number of *Lactobacillus* [64]. However, it is also associated with an increased number of *Escherichia coli* in the intestines which constitutes a potential risk factor for the intestinal disorders. Other medications, such as α-glycosidase inhibitor or liraglutide, can also affect the composition of the microbiota. In fact, the α-glycosidase inhibitor has demonstrated an inverse effect on the *Firmicutes* and *Bacteroidetes* ratio in the animal model [65]. On the other hand, liraglutide exerts a positive influence on the recombination of the intestinal microbiota which is associated with a better weight reduction. No effects of sitagliptin have been observed [66].

## 7. The Microbiota and Cardiovascular Diseases among Patients Suffering from Obesity

Cardiovascular disease (CVD) is a global public health concern and is one of the comorbidities of obesity. As the focus on the microbiota progresses, studies have shown that various microbial profiles and the gut microbiota-derived metabolites may also play a role in CVD development. Individuals with a history of CVD present higher fasting levels of trimethylamine-*N*-oxide (TMAO), and the production of TMAO is dependent on microbial metabolism, as a study by Tang et al. showed [67]. It is worth noting that TMAO can promote atherosclerosis and be also associated with heart failure [68,69]. Furthermore, it has been suggested that dysbiosis may potentially lead to hypertension (HT). In one study, increased *Firmicutes* and *Bacteroidetes* ratios and a decrease in the richness of the intestinal microbiota were observed among animal models; however, the results were confirmed in a small cohort of hypertensive patients in another study [70,71]. Dysbiosis is associated with a decrease in butyrate- and acetate-producing bacteria, as well as with an increase in the lactate-producing microbiota. According to Pluznick et al., acetate and propionate (SCFAs) produced by the intestinal microbiota influence blood pressure, which is partially mediated by olfactory receptor 78 [72]. In fact, bacteria, such as *Subdoligranulum, Ruminiclostridium, Intestinimonas, Pseudoflavonifractor, Paenibacillus,* and *Marvinbryantia*, could potentially prevent the development of hypertension [73]. It has been shown that atherosclerotic plaque hosts its microbiota, mainly consisting of *Proteobacteria*. Moreover, individuals with symptomatic atherosclerosis within the carotid artery showed higher levels of *Collinsella* when compared with the control group [74]. In addition, the gut hypothesis of heart failure is associated with decreased cardiac output, intestinal mesenteric ischemia, and oedema, leading to increased translocation of bacteria [68]. As mentioned earlier, increased permeability of the intestinal barrier is associated with increased systemic inflammation due to the translocation of several endotoxins or microbial metabolites (e.g., LPS). Taking the aforementioned factors into account, a “leaky gut” could support the progression of heart failure [75]. Therefore, investigating the association between the intestinal microbiota and cardiovascular disease could provide more knowledge regarding the pathogenesis of CVD, which, in turn, could lead to more effective management of the disease.

## 8. Diet, Nutritional Compounds, and Microbiota

Among other factors, diet—including dietary patterns and approaches, nutrients, and even food additives—plays a significant role in shaping and changing the microbiota composition. On the other hand, modulation of the intestinal microbiota through dietary changes could provide therapeutic and preventive actions against chronic diseases associated with dysbiosis. Nutrients can affect the microbiota in two ways (i.e., direct and indirect). The first one is associated with the promotion or inhibition of microbial growth. The other indirect effect is connected to the host organism’s immunologic and metabolic responses [76]. It is essential to remember that the effects of dietary changes in the composition of the intestinal microbiota can be seen after days, weeks, or even months. Moreover, various nutritional compounds can have an opposite or synergistic effect on the intestinal microbiota, rendering the association between certain types of diet or nutrients and the microbiota difficult to establish [77]. We have discussed several dietary patters that can affect the gut microbiota and are recommended or suggested for patients with obesity.

The Mediterranean diet (MeD) is a plant-based eating habit characterised by a high intake of vegetables, grains, legumes, fruits, olive oil, and red wine [78]. High adherence to the MeD was reportedly associated with the concentration of SCFAs in stool samples, with the highest association visible in vegetables, legumes, and fruit intake in [79]. On the other hand, lower adherence to the MeD was associated with higher urinary TMAO levels. Additionally, the association between TMAO levels and several microorganisms was found, and it was linked to the intake of fat and animal protein. A 2-year-long adherence to the MeD among patients with obesity resulted in an increase of several genera and species that were able to metabolise SCFAs from carbohydrates (Table 3) in [80]. Interestingly, the authors also suggested that due to higher phenolic and fibre content, MeD is more effective in restoring the gut microbiota functionality than a low-fat diet; nevertheless, a low-fat diet is also beneficial to the intestinal microbiota, although to a smaller extent. Moreover, it was found that a very low carbohydrate ketogenic diet (VLCKD) can modulate the intestinal microbiota, mainly if the contents of plant protein (reduced intake of animal protein), omega-3 fatty acids, fermented food, and beverages are high [81]. Additionally, plant protein increased *Bacteroidetes, Bifidobacterium*, and *Lactobacillus* while simultaneously decreasing *Firmicutes* in [82]. In mice, VLCKD significantly increased *Akkermansia* and *Parabacteroidetes,* and these changes mediated antiseizure effects in [83]. However, the influence of the VLCKD remains in some way controversial, and more research is necessary.

The previously mentioned Western-style diet (WsD) is a dietary pattern characterised by a high intake of animal protein, saturated fatty acids, total fat, and simple sugars, with a low intake of dietary fibre [77]. Nevertheless, it is vital to bear in mind that the WsD is also characterised by low cost, which leads to increased consumption, and further, it makes the WsD one of the main causes of noncommunicable diseases, such as obesity and dysbiosis, as recent studies have shown. High-fat Western-style diet consumption increases the number of several species (Table 3). These changes have been correlated with such disorders as cognitive impairments or inflammatory bowel diseases [84]. Additionally, the WsD often leads to an increased intestinal permeability and a decreased amount of SCFAs and enhances insulin resistance and inflammation. These results may seem contrary to studies that have demonstrated a positive effect of the VLCKD on the intestinal microbiota. However, it could be suggested that diet quality is more significant than the total intake of carbohydrates, protein, or in particular, fat. Saturated fatty acids should be avoided, while mono-unsaturated and poly-unsaturated fatty acid consumption should be increased in order to decrease the inflammatory state and weight gain associated with the impaired composition of the microbiota [85]. The influence of several diets, foods, and macronutrients on the intestinal microbiota is presented in Table 3. The identification of the link between microbial metabolites and metabolic diseases may be possible due to the isolation of several species and may explore the influence of micro- and macronutrients on their development [86].

Other nutrients, such as minerals, vitamins, and polyphenols, can also affect the intestinal microbiota—several studies have discussed their supplementation and intake in the composition of the gut microbiota. Polyphenols, which are not absorbed in the small intestine, move toward the large intestine, where the host microbiota metabolises them. It is worth noting that Gram-negative bacteria are more resistant to the effect of polyphenols than Gram-positive bacteria [87]. However, the specific interaction between polyphenols and the microbiota has not been fully investigated [88]. Studies indicate that the diet of T2DM patients rich in fibre, polyphenols, high-protein vegetables, and functional food, is in fact associated with lower *Prevotella copri* and an increase of anti-inflammatory bacteria: *Faecalibacterium prausnitzii* and *Akkermansia muciniphila* [89]. Additionally, vitamins, such as vitamin B2, B3, D, C, E, and A, as well as minerals, such as calcium, iron, potassium, zinc, and magnesium, can affect the intestinal microbiota. A summary concerning the influence of nutritional compounds on the composition of the intestinal microbiota is presented in Table 4.

## 9. FMT—When and for Whom?

There are indications suggesting that there are bacteria involved in the weight-gain process that can induce the expression of genes related to the metabolism of lipids and glucose and lead to an increased energy acquisition. The relationship between microorganisms and weight gain can be much more complex than simply due to an imbalance between different species of bacteria. Therefore, efforts have been made not only to use diets, probiotics, and prebiotics among obese patients but also to try other methods, such as faecal microbiota transplantation (FMT).

According to Kootte et al., in their over a 6-week-long study, FMT from lean individuals to individuals with diagnosed metabolic syndrome resulted in a significant reduction of HbA1c concentrations and led to improved insulin sensitivity in the study group. However, these results were not permanent, and following 18 months, the concentrations of HbA1c did not differ anymore [106]. Similar results were obtained from the study by Vrieze et al.; nevertheless, no changes in GLP-1, GIP, PYY, and lipid profile (mainly total cholesterol) were observed among patients who had undergone FMT [107]. The intestinal colonisation of various species can also affect body weight, as found in the previously mentioned study, where a reduction of body mass index (BMI) was observed. Subsequently, the study group was divided into individuals who observed FMT results and patients who did not, where a group of respondents reported increased levels of *muciniphila* in *Akkermansia*, as opposed to nonrespondents. In fact, *Akkermansia muciniphila* is associated with enhanced mucin degradation, and as studies on both humans and animals have shown, it is closely correlated with insulin sensitivity [108]. Its effects are presumably associated with increased concentrations of an endocannabinoid and epithelial toll-like receptor 2 responsible for intestinal function. In other studies, FMT resulted in the growth of 16 microorganisms, including butyrate-producing *Roseburia intestinalis* and *Clostridium* spp. [109]. Additionally, increased levels of oxylate-transforming *Oxalobacter formigenes* and *Clostridium* spp. were observed in the studied group as compared with the control group [110]. Since *Ruminococcus bromii* and *Roseburia intestinalis* are the species associated with the production of butyrate and the degradation of fibre [109], increasing their amount can play a role in insulin sensitivity improvement, as they regulate GLP-1 and are associated with the gluconeogenesis process in the intestine [111].

## 10. Conclusions

Obesity and its associated comorbidities have been connected with the altered intestinal microbiota. Moreover, current studies suggest that metabolic disturbances may begin in the intestines, since the gut microbiota-derived metabolites and gut dysbiosis can affect both the physiology and physiopathology of metabolic diseases. However, the problem is complex, with many significant coexisting factors, and requires further research. Although it should be investigated further, it is possible to conclude with high probability that the microbial aspect will provide an essential and novel tool in the understanding, treatment, and prevention of chronic diseases, including obesity. Furthermore, it can even be suggested that dysbiosis may be considered a new biomarker for metabolic disorders. Additionally, the gut–brain axis plays a crucial role in the host metabolism and appetite, which is connected to the prevalence of several metabolic disorders. Pharmacological and nutritional regulation of the intestinal microbiota could enhance and support the standard therapy for obesity and its comorbidities. Since nutrition constitutes an essential factor affecting the intestinal microbiota, we would suggest focusing on both the quality and the number of nutrients in order to address gut dysbiosis and metabolic disorders in a broader perspective.

Therefore, investigating gut crosstalk with the host could provide a new insight into metabolic disturbances and, consequently, could lead to new treatment approaches.

## Figures and Tables

**Table 1:** The results of the meta-analysis of Million et al. [33].

An Increase in Body Weight	A Decrease in Body Weight
*Lactobacillus acidophilus*	*Lactiplantibacillus plantarum*
*Limosilactobacillus fermentum*	*Lactobacillus gasseri*
*Lactobacillus ingluviei*	

**Table 2:** The probiotic species beneficial for body weight reduction.

**Species Supporting Body Weight Reduction**
*Ligilactobacillus salivarius*
*Lacticaseibacillus paracasei*
*Lactobacillus gasseri*
*Limosilactobacillus reuteri*
*Bifidobacterium lactis*

**Table 3:** The effect of several diets/macronutrients on the intestinal microbiota composition.

Favourable Effect on the Intestinal Microbiota Influence on the Gut Microbiota *	Adverse Effect on the Intestinal Microbiota Influence on the Gut Microbiota *
Mediterranean Diet	Western-style diet
*Bacteroidetes* ↑, *Faecalibacterium* ↑, *Prevotella, Ruminococcus* ↑, *Roseburia*, *Parabacteroides distasonis* ↑, *Faecalibacterium prausnitzii* ↑	*Firmicutes* ↑, mollicutes ↑, *Clostridium* ↑, *Enterobacteriaceae* ↑, *Bilophila* ↑
Very low carbohydrate ketogenic diet?	Animal protein
Plant protein *Bacteroidetes* ↑, *Bifidobacterium* ↑, *Lactobacillus*, *Firmicutes* ↓	Saturated fatty acids
Fibre	Total fat intake
Fermented food	Simple sugars
Fermented, nonalcoholic beverages	

* Only selected studies on humans are included. ↑—an increase in the number of specific bacteria, ↓—a decrease in the number of specific bacteria.

**Table 4:** The influence of nutritional compounds on the composition of the intestinal microbiota.

Nutritional Compound	Possible Changes in the Microbiota	Model	Type of Determining Compositional Changes	Comment
Minerals				
Potassium [90]	*Bacteroidetes ↓*	Adults with cystic fibrosis	16S rDNA seq	
Calcium [91,92]	*Lacticaseibacillus paracasei ↑*	Adults	qPCR	Calcium supplementation (pentacalcium hydroxy-triphosphate)
	*Bifidobacterium spp*,	Male mice	qPCR	HF diet compared with HCa diet (4 g/kg vs. 12 g/kg of calcium)
	*Bacteroides/Prevotella ↑*			
	*Clostridium coccoides ↓,*			
	*Clostridium leptum ↓*			
Iron [93,94]	*Enterobacteria ↑, Lactobacilli ↓*	Anaemic African children	PCR and temporal temperature gradient electrophoresis analyses	Iron-fortified biscuits
	*Bifidobacterium↑*	Japanese people	T-RFLP method	Habitual diet
Magnesium [95]	*Bifidobacteria ↓ (LI)* Diversity *↓*	Mg-deficient mice	Real-time quantitative PCR	fed a control or Mg-deficient
Zinc [96]	*Proteobacteria ↑*	Chicks	16S rRNA PCR	Diets with various content of Zn
	*Firmicutes↓*			
	*Bacteroidetes ↑*			
	Actinobacteria ↓ *			
Polyphenols				
Chokeberry extract [97]	*Anaerostipes ↑*	Adult men	16S rRNA seq	
Chokeberry whole fruit [97]	*Bacteroidetes ↑*			
Red wine [98]	*Proteobacteria ↑,*	Adult men	qPCR	4-week-long consumption
	*Fusobacteria ↑,*			
	*Firmicutes,*			
	*Bacteroidete ↑*			
Alcohol-free wine [98]	*Fusobacteria*,			4-week-long consumption
	*Bacteroidetes ↓*			
	*Firmicutes ↓*			
Tea [99]	*Clostridium perfringes (IH)*	Collected human faeces	(measure of growth) 5% inoculum, the optical density anaerobic conditions	Tea phenolics and metabolites
	*Clostridium difficile (IH)*			
	*Bacteroidetes (IH)*			
Catechins [100]	*Clostridium cocoides ↑*	Collected human faeces	16S rRNA seq	
	*Bifidobacterium ↑*			
	*Escherichia coli ↑*,			
	*C. histolyticum (IH)*			
Gallocatechin [90]	*Actinomyces ↑*, *Actinomycetaceae ↑*, *Coriobacteria ↓*	Adults with cystic fibrosis	16S rDNA seq	
Vitamins				
Vitamin A [101,102]	*Bacteroidetes vulgatus ↓*	Mice	16S rDNA seq	LI of vitamin A
	*(supp.) Bacteroidetes ↑*	Children with ASD	16S rDNA seq	Vitamin A supplementation
	*(supp.) Firmicutes ↓*			
	*(supp.) Proteobacteria ↓*			
	*(supp.) Actinobacteria ↓*			
	*Bifidobacterium ↑*			Observed only among boys
Vitamin D [103,104]	*Veillonella ↑*	Adults with CF	16S rRNA seq	Vitamin D supplementation
	*Lactococcus ↑*			
	*Erysipelotrichaceae ↑*			
	*Lachnospira ↑*	Adults	16S rRNA seq	Vitamin D supplementation
	*Blautia ↓*			
	*Ruminococcus ↓*, *(supp.)*			Serum concentrations of 25(OH)D > 75 nmol/L were associated with higher number when compared with concentration 25(OH)D < 50 nmol/L
	Coprococcus *↑*,			
	*Enterobacteriaceae ↑*	Adults with UC	16S rRNA seq	Vitamin D supplementation
Vitamin C [105]	*Coriobacteriaceae ↑*	Adults with prediabetes	16S rRNA seq	Consumption of two SunGold kiwifruit
Vitamin E Niacin Riboflavin [90]	*Firmicutes ↑*	Adults with cystic fibrosis	16S rDNA seq	

CF—cystic fibrosis; HF—high-fat diet; HCa—high-calcium diet; LI—low intake; supp.—supplementation; IH—inhibition of the growth; ↑—an increase in the number of specific bacteria; ↓—a decrease in the number of specific bacteria; UC—ulcerative colitis; T-RFLP—terminal restriction fragment length polymorphism; PCR—polymerase chain reaction; *—nonsignificant; ASD—autism spectrum disorder.

## Data Availability

MDPI Research Data Policies.
